# EPIGENETIC AGING IN THE HRS

**DOI:** 10.1093/geroni/igz038.1613

**Published:** 2019-11-08

**Authors:** Jessica Faul

**Affiliations:** University of Michigan, Ann Arbor, Michigan, United States

## Abstract

Biological aging can be characterized by molecular, cellular, and epigenetic changes that in addition to being related to chronologic age, are also associated with social disadvantage and associated morbidity and mortality. These biological markers can help explain at a biological level why socially disadvantaged individuals are at greater risk of aging-related disease and premature death. From DNA extracted from venous blood collected from over 4,000 HRS participants we measured array based DNA methylation. These assessments were made from unsorted cells, but are adjusted for individual cell composition measured from flow cytometry. We present genome-wide methylation differentials by age, race/ethnicity and SES using the largest, nationally representative sample with these data available to date. Understanding basic biological changes related to age and social disadvantage is essential for identifying translational opportunities to improve health.

